# Decentralized surgery of abdominal wall defects in Germany

**DOI:** 10.1007/s00383-020-04647-7

**Published:** 2020-03-26

**Authors:** Andrea Schmedding, Boris Wittekind, Emilia Salzmann-Manrique, Rolf Schloesser, Udo Rolle

**Affiliations:** 1 Department of Pediatric Surgery and Pediatric Urology, University Hospital, Goethe University Frankfurt, Theodor-Stern-Kai 7, 60590 Frankfurt am Main, Germany; 2 Department of Neonatology, University Hospital, Goethe University Frankfurt, Theodor-Stern-Kai 7, 60590 Frankfurt am Main, Germany; 3 Department of Pediatric Stem Cell Transplantation, University Hospital, Goethe University Frankfurt, Theodor-Stern-Kai 7, 60590 Frankfurt am Main, Germany

**Keywords:** Gastroschisis, Omphalocele, Neonatal surgery outcome, Child, Mortality

## Abstract

**Purpose:**

Neonatal surgery for abdominal wall defects is not performed in a centralized manner in Germany. The aim of this study was to investigate whether treatment for abdominal wall defects in Germany is equally effective compared to international results despite the decentralized care.

**Methods:**

All newborn patients who were clients of the major statutory health insurance company in Germany between 2009 and 2013 and who had a diagnosis of gastroschisis or omphalocele were included. Mortality during the first year of life was analysed.

**Results:**

The 316 patients with gastroschisis were classified as simple (82%) or complex (18%) cases. The main associated anomalies in the 197 patients with omphalocele were trisomy 18/21 (8%), cardiac anomalies (32%) and anomalies of the urinary tract (10%). Overall mortality was 4% for gastroschisis and 16% for omphalocele. Significant factors for non-survival were birth weight below 1500 g for both groups, complex gastroschisis, volvulus and anomalies of the blood supply to the intestine in gastroschisis, and female gender, trisomy 18/21 and lung hypoplasia in omphalocele.

**Conclusions:**

Despite the fact that paediatric surgical care is organized in a decentralized manner in Germany, the mortality rates for gastroschisis and omphalocele are equal to those reported in international data.

## Introduction

Gastroschisis and omphalocele are rare diseases, but are the most common abdominal wall defects. The prevalence of gastroschisis is 4–5 per 10,000 births, and that of omphalocele is 2 per 10,000 births [1, 2]. Gastroschisis is an abdominal wall defect in which the abdominal organs eviscerate through an opening in the abdominal wall that is usually located on the right side of the umbilicus [3, 4]. The malformation can be classified as complex, defined by the presence of intestinal atresia, perforation, necrotic segments, or volvulus, or as simple; the category of the defect has an impact on the outcome [5]. With omphalocele, herniation of organs into the umbilical cord occurs. Infants with omphalocele usually present with a hernia sac. Omphalocele is more often accompanied by associated anomalies, especially chromosomal anomalies [3, 4].

Abdominal wall defects are usually detected using prenatal ultrasound [6, 7]. This allows prenatal referral to a centre with experience in paediatric surgery, neonatology and specialized obstetrics for most women. In Germany in 2005, the joint federal committee (G-BA) decided on regulations regarding the quality of care for preterm and newborn babies in perinatal centres. These regulations divided prenatal care into levels; level one indicates the highest level of care, which means the care of neonates born at less than 29 weeks of gestation or birth weight less than 1250 g. For these units, it is necessary to have regulated access to paediatric surgery [8]. In keeping with the growing number of level 1 units after 2005, the number of paediatric surgical units increased as well. As the number of births and the number of children with congenital malformations did not increase in parallel, we now have a system that includes small specialized neonatal units [9] and a small caseload for congenital malformations, as could be shown for the year 2015, in which 89 departments of paediatric surgery treated 93% of the abdominal wall defects with an average case load of less than five cases per unit [10].

Because of the historical development towards decentralization of paediatric surgery in Germany, the question arose whether the outcome of congenital wall defects in Germany is equal to the outcomes reported in international results. In Germany, no clinical national registries exist for these congenital deformities.

A hospital treatment leads to a dataset that is provided to the health insurance companies for accounting purposes; this dataset includes diagnoses and procedures. Data of this type have been used in the analysis of clinical questions [11]. The dataset of the largest health insurance company was used for the analysis of the treatment and outcome of abdominal wall defects.

## Methods

The database of the major health insurance company in Germany (Allgemeine Ortskrankenkasse, AOK) was retrospectively analysed for the years 2009–2013. This database is produced for accounting purposes according to §301 of the German social security code V. It contains personal and medical data of the patients. It includes codes for diagnoses based on the International Statistical Classification of Diseases and Related Health Problems—German modification (ICD-10-GM) [12] and codes for procedures based on the German procedure classification (OPS) [13]. For each medical dataset one main diagnosis, up to 20 secondary diagnoses and 99 procedures can be submitted from the hospitals to the insurance company [14].

In Germany in 2011, 87% of the inhabitants were insured under statutory health insurance (SHI) [15, 16]. The AOK is the largest statutory health insurance company in the country; it has branches all over the country and covers approximately one-third of German patients. Regarding the socioeconomic status of the clients of AOK, the microcensus of Germany conducted in 2011 (Table 1) shows that the percentage of jobless persons and others who are not working (e.g. children, retired persons) is slightly higher among the insured persons of AOK than among people insured through other insurance companies [17]. Additional factors regarding the socioeconomic status of the insured people were analysed in 2012, and they also indicated a lower socioeconomic status of insured people at AOK [18]. This must be taken into consideration when analysing the data obtained from AOK.

**Table 1:** Employment status and age under 15 years for people with health insurance

2011 microcensus of Germany	Employed	Jobless	Not working (e.g. children, retired)	Age less than 15 years
All people with health insurance	52%	3%	48%	13%
All people in statutory health insurance	48%	3%	49%	13%
AOK	41%	5%	54%	13%

The sample size estimation was made using the statistics for live births in Germany [19]. Taking the number of live births in Germany as 670,000, the prevalence of gastroschisis as 4:10,000 and that of omphalocele as 2:10,000, the expected number of patients with gastroschisis (G) was 268 per year, and the expected number of patients with omphalocele (O) was 134 per year. This would lead to 89 (G) and 45 (O) patients in the AOK database per year. Therefore, an analysis of five consecutive years was made.

All newborn patients who were clients of AOK between 2009 and 2013 with a diagnosis of gastroschisis or omphalocele at first admission to the hospital were identified by the ICD-10 codes Q79.3 for gastroschisis and Q79.2 for omphalocele either as the main code or as a secondary code. The full sample of data was used; no patients were withdrawn from the study.

For each patient, the following parameters were obtained and analysed: year of admission, level of perinatal centre, paediatric surgery at the perinatal centre, target diagnosis, gender, birth weight less than 1500 g, length of primary hospital stay, alive at age of 30 days, 3 months, 6 months and 12 months, and secondary diagnoses (K55: abnormalities of vascular supply of intestine, K56.2: volvulus, Q41: atresia of small bowel, Q42: atresia of colon, Q20-24: cardiac anomalies, Q60-64: anomalies of urinary tract, Q76: anomalies of spine or thorax, Q79.0: diaphragmatic hernia, Q33.6: lung hypoplasia, Q90-91: trisomy 18 or 21). The following procedures (OPS) were documented if performed: 5-537, closure of congenital defects of abdominal wall; and 5-431, gastrostomy. The birth weight, which is a mandatory parameter required by the insurance companies, was either taken from the ICD Code P07.00-P07.11 (birthweight less than 1500 g) and/or the weight itself. Data for follow-up were obtained at 3 months, 6 months and 12 months and included the parameters of survival and the number of new hospital admissions.

For further analysis, a detailed list of the 50 most frequent diagnoses and procedures coded for these patients was also compiled.

### Statistical methods

Descriptive statistics are presented as frequencies and percentages or median with quartiles and range for categorical and continuous variables, respectively. Categorical variables were compared using Fisher’s exact test or the Chi-square test.

For the analysis of the length of hospital stay (LOS), we used the nonparametric cumulative incidence function of Fine and Gray [20]. The cumulative incidences of LOS for patients who were discharged to home were performed treating the in-house mortality as a competing risk [21].

In our data, the precise time of death after discharge is unknown. However, all alive patients were followed up for longer than 12 months after discharge, and the status of all patients (alive, died) is known at this time point. Therefore, we used logistic regression to identify variables associated with death from birth to 1 year later. To assess the impact of the variables, odds ratios (ORs) with 95% confidence intervals (95% CI) were calculated. Multivariate analysis was not possible for the gastroschisis cohort due to the low number of non-survivors. We adjusted the odds ratio considering the variables sex, birth weight, cardiac anomalies, lung hypoplasia and trisomy 18/21 in the omphalocele patients group.

All tests were two-sided, and a *P* value < 0.05 was considered statistically significant. Analyses were performed using R statistical software version 3.5.3 [22].

## Results

We identified 513 patients, 316 with gastroschisis (G) and 197 with omphalocele (O). All, but 12 patients with gastroschisis (96%) and all but 3 patients with omphalocele (98%) were treated at perinatal centres providing paediatric surgical treatment or separate paediatric surgical departments. Gender was documented in all but two patients with gastroschisis and in all but four patients with omphalocele. The male:female ratio was 53:47% in gastroschisis and 58:42% in omphalocele. The distribution of the patients, which was rather consistent over the 5-year period, is shown in Table 2.

**Table 2:** Distribution of diseases over the 5-year period

	All	Male	Female	BW < 1500 g	Non-survivor
Gastroschisis					
2009	61	36	25	6	3
2010	69	36	33	3	2
2011	63	28	34	5	3
2012	55	29	26	2	3
2013	68	37	30	5	1
All	316	166	148	21	12
%		53%	47%	7%	4%
Omphalocele					
2009	34	14	20	3	4
2010	42	28	14	0	7
2011	42	24	17	5	8
2012	46	25	20	4	6
2013	33	20	11	3	7
All	197	111	82	15	32
%		58%	42%	8%	16%

*BW* birth weight

Associated anomalies are documented in Table 3. Complex gastroschisis was defined as gastroschisis together with atresia of the small bowel (Q41), atresia of the colon (Q42), volvulus (K56.2), or problems with the vascular supply to the intestine (K55) according to Arnold [4]. Perforation and necrotic segments were not indicated in the dataset. The latter was assumed when the diagnosis of abnormal vascular blood supply was documented. Complex gastroschisis was found in 58 children, 17% of whom had birth weight below 1500 g, in contrast to 4% with simple gastroschisis. The male:female ratio was opposite in complex gastroschisis, with a female predominance of 61%. Intestinal problems that define complex gastroschisis were present in 7% of patients with omphalocele. Only one of these patients had birth weight below 1500 g. The male:female ratio was 46:54%.

**Table 3:** Distribution of related associated anomalies and relation to survival

	Gastroschisis		Omphalocele	
	All (%)	Non-survivors (%)	All (%)	Non-survivors (%)
Numbers	316	12	198	32 (16)
Male	166 (53)	4 (33)	111 (58)	10 (31)
Birth weight < 1500 g	21 (7)	4 (33)	15 (8)	8 (25)
Intestinal problems^a^	58 (18)	6 (50)	13 (7)	3 (9)
Atresia of small bowel	35 (11)	3 (25)	6 (3)	0
Atresia of colon	22 (7)	2 (17)	4 (2)	1 (3)
Volvulus	8 (3)	2 (17)	1 (1)	0
Abnormal vascular supply of intestine	13 (4)	3 (25)	3 (2)	2 (6)
Cardiac anomalies	32 (10)	0	63 (32)	17 (53)
Lung hypoplasia	1 (< 1)	1 (8)	9 (5)	7 (22)
Diaphragmatic hernia	0	0	4 (2)	2 (6)
Anomalies of urinary tract	17 (5)	0	19 (10)	5 (16)
Anomalies of spine or thorax	0	0	3 (2)	1 (3)
Trisomy 18 or 21	0	0	15 (8)	13 (41)

Procedures and follow-up	All (%)	n.d.a.	All (%)	n.d.a
CAW	235 (74)		144 (73)	
Appendectomy	43 (14)		10 (5)	
Blood transfusion	126 (40)		68 (34)	
Gastrostomy	5 (2)		11 (6)	
Closure of ing. hernia			11 (6)	
Short bowel	16 (5)			

Survivors	Median (average)	IQR (range)	Median (average)	IQR (range)
Length of initial stay	39 (61.0)	28–69 (15–1069)	19 (41.6)	11–35.5 (3–285)
Simple gastroschisis	35 (47.0)	27–52.25 (15–272)		
Complex gastroschisis	105.5 (128.0)	47.75–149.75 (18–1069)		
No. of readmissions	1 (1.2)	0–2 (0–11)	0 (1.4)	0–2 (0–9)
Simple gastroschisis	1 (1.0)	0–2 (0–8)		
Complex gastroschisis	2 (2.3)	1–3 (0–11)		

*n.d.a.* no data available, *CAW* closure of abdominal wall without synthetic material, *IRQ* interquartile range

^a^In gastroschisis correspondent with complex gastroschisis

Urologic anomalies were more frequent in patients with omphalocele (10% O, 5% G), as were cardiac anomalies (32% O, 10% G). Fifteen patients with omphalocele had trisomy 18 or 21, and 12 had both trisomy and cardiac anomalies. Diaphragmatic hernia was present in 2% of the patients with omphalocele. Short bowel was coded for patients with gastroschisis only (5%).

Mortality was 4% in the gastroschisis group (12 patients) and 16% in the omphalocele group (32 patients). Half of the non-survivors with gastroschisis and 44% of the non-survivors with omphalocele died during the first 10 days. Of the non-survivors with gastroschisis and omphalocele, 25% died after the third month of life. Survivors had a median length of stay at the first admission of 39 days (G) and 15 days (O) with ranges of 15–1069 days (G) and 3–285 days (O). The average initial hospital stay of patients with complex gastroschisis was 128.0 days (median 106). Readmissions were also more frequent in patients with complex gastroschisis and omphalocele than in those with simple gastroschisis. Of the survivors with gastroschisis, 61% had no readmission at the 3-month follow-up, and this decreased to 41% at the 12-month follow-up. Of those with omphalocele, 58% had no readmission at the 3-month follow-up, and this also decreased to 41% at the 12-month follow-up. The maximum number of readmissions during the first 12 months was 11 for patients with gastroschisis and 9 for patients with omphalocele.

Closure of the abdominal wall without synthetic material and without temporary closure could be performed in 74% of the patients with gastroschisis and 73% of those with omphalocele. In 43 (G) versus 10 (O) patients, appendectomy was performed. Five patients with gastroschisis and 11 with omphalocele required gastrostomy during the first 12 months, and 6% of the patients with omphalocele had closure of inguinal hernia during the first stay. Blood transfusion was required in 40% of the patients with gastroschisis and 34% of the patients with omphalocele.

The following variables were assessed for their impact on death from birth to 1 year later: sex, birth weight < 1500 g, intestinal problems including atresia of the small bowel, atresia of the colon, volvulus and abnormal vascular supply to the intestine, cardiac anomalies, lung hypoplasia, anomalies of the urinary tract and trisomy 18 or 21.

**Fig. 1:**
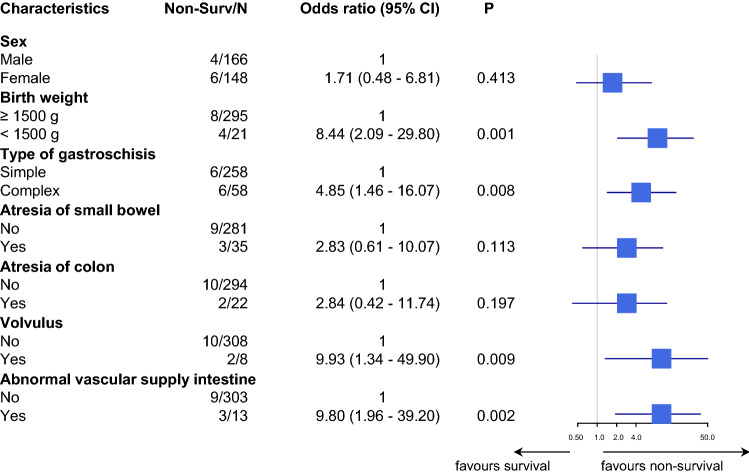
Gastroschisis: association with non-survival the first year of life. Forest plot shows the unadjusted odds ratio in base-10 log scale. *Non-Surv* non-survivors, *CI* confidence interval, *Pp* value

**Fig. 2:**
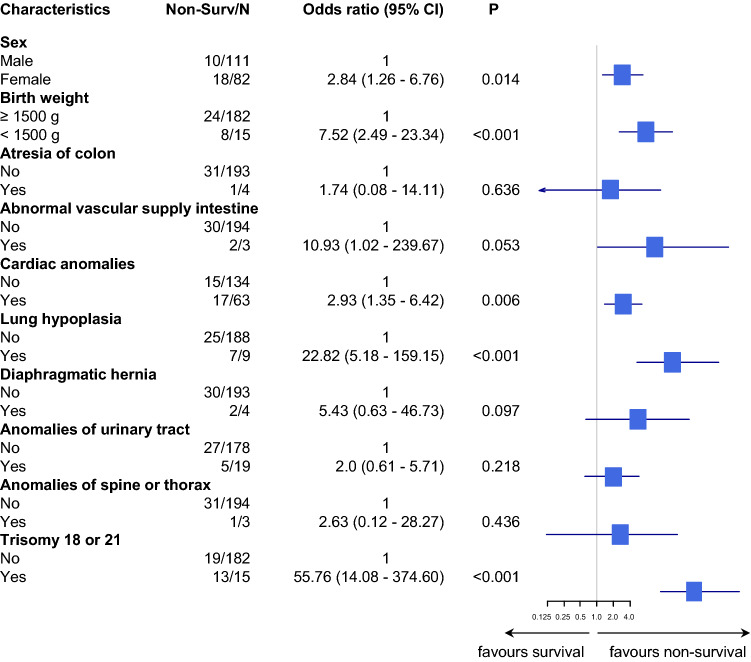
Omphalocele: association with non-survival during the first year of life. The forest plot shows the unadjusted odds ratio on a base-10 log scale. *Non-Surv* non-survivors, *CI* confidence interval, *Pp* value

In both groups, birth weight below 1500 g was associated with significantly worse clinical outcome. The odds ratios (95% CI) of patients with birth weight above 1500 g were 8.44 (2.09–29.80) and 7.52 (2.49–23.34) compared to patients with birth weight ≥ 1500 g in the gastroschisis group and in the omphalocele group, respectively (Figs. 1, 2).

**Fig. 3:**
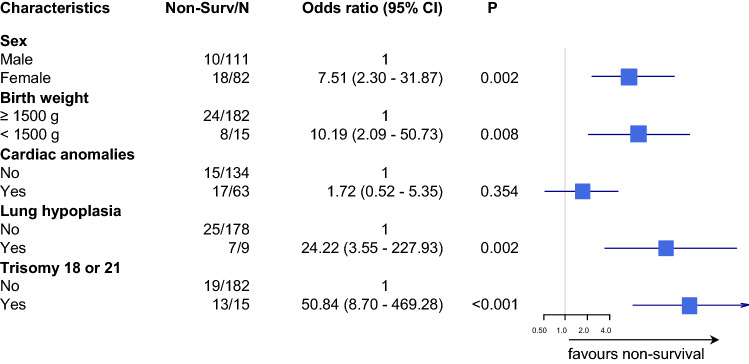
Omphalocele: forest plot showing the adjusted odds ratio associated with non-survival in the first year of life on a base-10 log scale. *Non-Surv* non-survivors, *CI* confidence interval, *Pp* value

In the gastroschisis group, intestinal problems had a negative effect on survival. This was significant for complex gastroschisis (odds ratio 4.85; 95% CI 1.46–16.07), volvulus (odds ratio 9.93; 95% CI 1.34–49.90) and abnormal blood supply of the intestine (odds ratio 9.80; 95% CI 1.96–39.20) (Fig. 1). In the omphalocele group, female gender, lung hypoplasia and trisomy 18 and 21 had a significant negative impact on survival (Fig. 2). In the adjusted model, lung hypoplasia (odds ratio 24.22; 95% CI 3.55–227.93) and trisomy 18 and 21 (odds ratio 50.8; 95% CI 8.70–469.28) remained significant, while cardiac anomalies lost significance (Fig. 3).

**Fig. 4:**
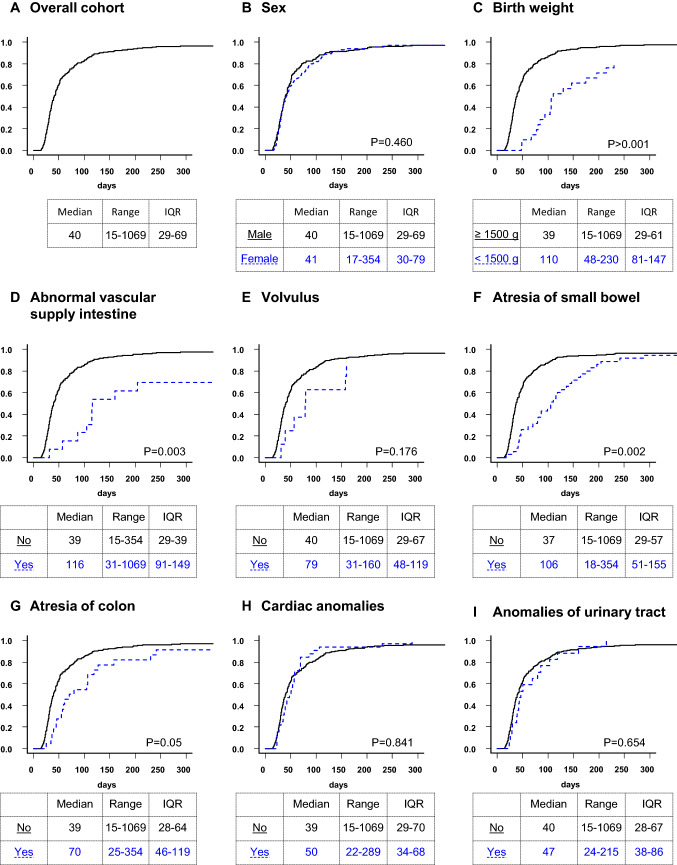
Gastroschisis. Cumulative incidence curves for hospital discharge. The curves do not reach 100% because fatalities before discharge were considered competing risks

**Fig. 5:**
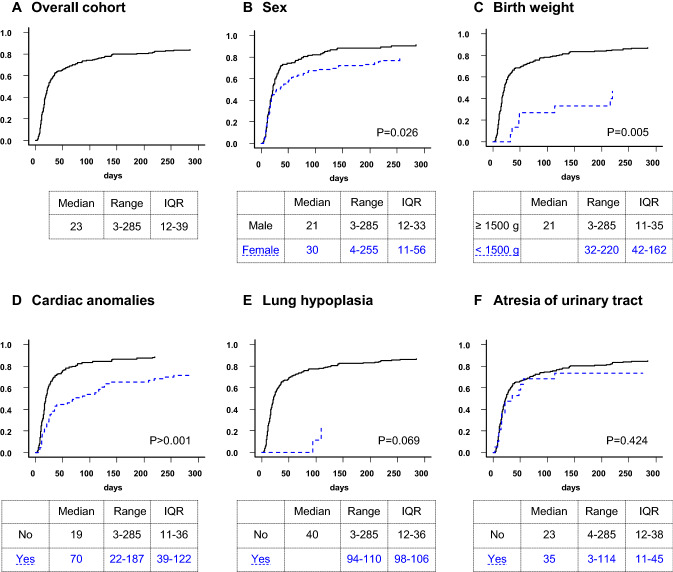
Omphalocele. Cumulative incidence curves for hospital discharge. The curves do not reach 100% because fatalities before discharge were considered competing risks. It was not possible to calculate a median for omphalocele patients with lung hypoplasia, and trisomy 18 and 21 because 78% and 87% of the patients died before discharge

Figures 4 and 5 present the length of hospital stay for patients who were discharged home. In the gastroschisis group, no difference in the length of stay was found regarding sex, cardiac anomalies, volvulus and anomalies of the urinary tract. In contrast, birth weight, abnormalities of vascular supply of the intestine, atresia of the small bowel and atresia of the colon were associated with a significantly longer hospital stay. In the omphalocele group, patients with birth weight below 1500 g as well as cardiac anomalies and female patients showed a significantly longer hospital stay.

## Discussion

Congenital anomalies are the second most prevalent cause of infant deaths in Germany [23]. Analysis of the medical care of patients with rare congenital anomalies is challenging in a country such as Germany, in which no nationwide clinical registry exists. Data that were originally collected for financial purposes have been shown to be a reliable resource for epidemiologic studies [11, 24, 25]. The strength of our study lies in the large cohort of approximately one-third of the German patients; this is the largest number of patients that has been analysed in a single study in Germany. But there are some differences regarding the population of persons insured by other statutory health insurance companies in terms of the age structure and comorbidity profile and the fact of a lower socioeconomic status of insured people at AOK [18].

As we used insurance claims data, there are some limitations in the study design. In Germany in 2003, the billing system for hospitals was changed to diagnosis-related groups (DRG). Hospitals receive a fixed income per treatment case based on the DRG of each case. For the assignment of DRG, patient-related data as ICD codes, age and procedures are used. Secondary diagnoses can lead to a better financed DRG, but not all do. The same applies to procedures. Together with the limitation on the number of secondary diagnoses to 20 per case, there might be an underestimation of associated anomalies that do not trigger a DRG.

We estimated the number of patients based on the birth statistics for Germany. The number of patients received from the AOK claims database was approximately 30% lower for gastroschisis and 10% lower for omphalocele than the estimated number with incidence taken from existing literature [1, 2]. This is due to the fact that the real incidence of gastroschisis and omphalocele is not known in Germany. There are only two regional registries for the epidemiology of congenital anomalies (Mainz and Saxony-Anhalt), covering about 3% of Germany. In these registries, the prevalence of gastroschisis was 3.06 per 10,000 livebirths, and the prevalence for omphalocele 0.69 per 10,000 livebirths for the years 2009–2013 [26]. With these data the estimated number of gastroschisis would nearly fit to our data (6% lower than estimated), but not for omphalocele (156% higher than estimated). Another reason that the prevalence of gastroschisis is lower than our estimation from literature is that gastroschisis is linked to low maternal age [27]. In Germany, the mean maternal age at first birth was 28.8 years in 2009, and it increased to 29.3 years in 2013 [28]. Therefore, the prevalence of gastroschisis might be lower in Germany than in other countries with lower maternal age at pregnancy. The other cause of lower prevalence could be the termination rate. Gastroschisis and omphalocele are usually diagnosed prenatally [7]. In the Netherlands, an improved prenatal detection rate increased the pregnancy termination rate for abdominal wall defects [29]. A similar effect may apply to Germany, but no valid data on this are available.

Nevertheless, our data fit to other series described in the literature, as shown in Table 4. For both entities, we found male predominance, but male predominance is lower in gastroschisis than in omphalocele. The distribution of associated anomalies is consistent with that reported in the literature.

**Table 4:** Comparison of our data with literature

	Gastroschisis		Omphalocele	
	Our data	literature^a^	Our data	literature^a^
Male (%)	53	52–54	58	51–59
< 1500 g (%)	7	0.1–5.6	8	6–8
Cardiac (%)	10	1–15	32	11–56
Pulmonary (%)	< 1	< 1–5	5	2–8
Gastrointestinal (%)	18	5–15	7	7–15
Urologic (%)	5	1–15	10	6–17
Genetic (%)	0	0.1–1.7	8	15–32
Length of stay (median)	39	25–45	15	15–23
Mortality (%)	4	1,4–6,5	16	16–19

^a^[24, 30, 31, 34–41]

In gastroschisis, the predominant anomalies derive from the gastrointestinal tract. In our series, 18% of the cases could be classified as complex, showing intestinal atresia, volvulus or anomalies of vascular supply of the intestine; this is consistent with a previously reported series [30, 31]. The incidences of colonic atresia and small bowel atresia are almost equal, which is fits to the series of Fleet and Shah [32, 33]. We found cardiac anomalies in one-third of the patients with omphalocele. The incidence of genetic anomalies was lower than that reported in the literature, and this might be due to prenatal counselling and termination of pregnancies.

In our group, gastroschisis has a low mortality rate of 4%. This is slightly higher than the best rates reported in the literature, but we also found a higher rate of children with birth weight under 1500 g, a condition that has a negative impact on survival. In other studies, these children were not counted [41]. Consistent with the literature, we confirmed that complex gastroschisis has an impact on the outcome, resulting in longer hospital stay and higher mortality [40]. The rate of short bowel syndrome in gastroschisis is 5%, almost the same as Raymond found for a larger series in the USA (6%) [40]. The outcome in patients with omphalocele is mainly determined by the presence of trisomy, low birth weight or lung hypoplasia. Mortality in patients with omphalocele is four times as high as in patients with gastroschisis. This is also consistent with other series. In both groups, intestinal atresia alone had no significant impact on survival. The longer hospitalization of patients with gastroschisis than of those with omphalocele has been shown in other series as well [34]. The reason for this cannot be determined based on our data, because the data do not contain information on the course of enteral feeding as one of the possible reasons for delayed discharge. Further research based on clinical data is needed here.

The debate regarding centralization of paediatric surgery is an ongoing discussion. Between 2009 and 2013, about 110 paediatric surgical units were registered in Germany [10]. Due to data protection requirements, our study does not reveal information on the centres, which treated abdominal wall defects. In an earlier study, we showed that paediatric surgery is organized in a decentralized way in Germany for legislative and historical reasons. This effect is homogeneous for our country. In that study, only one unit had performed more than 15 procedures for closure of abdominal wall defects [10]. For this reason, we took Germany as an example for low-volume (1–4 cases per year) and medium-volume (5–13 cases per year) hospitals according to the classification of Dubrovsky [35].

Based on our data, we showed that the outcome of gastroschisis and omphalocele is equal to the outcome reported in international series. Therefore, we conclude that there is no negative volume effect on mortality, similar to the recent conclusions of Dubrovsky and Hong [35, 41].

## Conclusion

Despite the fact that paediatric surgical care is organized in a decentralized manner in Germany, the mortality rates for gastroschisis and omphalocele are equal to those reported in international data.
